# Validating a remote saliva collection tool for genomic analyses in free ranging dogs

**DOI:** 10.1038/s41598-025-19267-1

**Published:** 2025-10-17

**Authors:** Giulia Cimarelli, Martina Lazzaroni, Clément Car, Ikhlass El Berbri, Sarah Marshall-Pescini, Małgorzata Pilot

**Affiliations:** 1 Domestication Lab, Konrad Lorenz Institute of Ethology, University of Veterinary Medicine Vienna, Savoyenstrasse 1a, Vienna, 1160 Austria; 2 Behavioural Ecology Group, Wageningen University and Research, De Elst 1, Wageningen, 6708 The Netherlands; 3 Department of Chemistry, Life Science and Environmental Sustainability, University of Parma, Viale delle Scienze 17/A, Parma, 43124 Italy; 4 Faculty of Biology, University of Gdańsk, ul. Wita Stwosza, Gdańsk, Poland; 5 Department of Veterinary Pathology and Public Health, Agronomy and Veterinary Institute Hassan II, Rabat, Morocco

**Keywords:** DNA yield, Free-ranging dogs, Genomic analyses, Non-invasive sampling, Saliva collection, Wildlife genetics, Behavioural methods, Genomic analysis

## Abstract

Saliva is a well-established source of DNA for various applications due to its non-invasive collection and its provision of high-quality DNA. However, its use in wild and free-ranging animal research remains limited due to challenges in collection without direct animal handling. In this study, we developed and evaluated a hands-off saliva collection method designed for free-ranging domestic dogs (FRDs), serving as a model for non-invasive genetic sampling of wildlife. Our method utilized a funnel paired with a commercially available Performagene kit (DNA Genotek, Canada), presented to the dog in the presence of an operator. The dog was free to approach and interact with the apparatus, depositing saliva while trying to reach bait. We compared DNA yield and genotyping success from samples using this hands-off method with those collected via the manufacturer’s recommended method. We collected 461 saliva samples from 326 FRDs, performing 750 DNA extractions. Samples collected by hand yielded significantly higher DNA concentrations after the first extraction attempt (mean = 46.3 ng/µL) than those collected using the hands-off method (mean = 32.2 ng/µL). Despite lower DNA concentrations, genotyping success did not significantly differ between methods, demonstrating that the hands-off method can yield DNA suitable for genomic analyses. The hands-off saliva collection method is a viable alternative to invasive sampling, addressing ethical concerns and enabling genomic studies in wild animals. Furthermore, our method mitigates sampling bias toward bold individuals, a common limitation in behavioral and genetic studies of free-ranging animals. With minor adaptations, this method could be applied across various species, including more elusive ones, contributing to conservation genetics and behavioral ecology research.

**Supplementary Information:**

The online version contains supplementary material available at 10.1038/s41598-025-19267-1.

## Introduction

Since the introduction of the DNA collection method from saliva at the beginning of the 21st century^1,2^, saliva has become a well-established and widely used source of DNA for multiple applications in research and medical diagnostics^3,4^. The advantage of saliva sampling over other DNA sources is its non-invasive character and thus the ease of sampling, combined with the superior quality and quantity of DNA compared with other non-invasive DNA sources such as buccal swabs, hair or faeces. These qualities have led to salivary DNA being increasingly used as an alternative to blood samples in biomedical and veterinary research^5,6^.

The same qualities make saliva a potentially important DNA source for research and monitoring of wildlife and free-ranging domestic animals. Such studies frequently involve invasive procedures, such as blood sampling, tissue biopsies, toe-clipping in small vertebrates, resulting in a conflict between the need to obtain information about the study populations and individual animal welfare^7^. Broader implementation of non-invasive sampling techniques could reduce this conflict, but despite intensive development of such techniques^8,9^, studies implementing them are still a minority in wildlife research^10^. This may be because most non-invasive sources of DNA, e.g. faeces, urine, hair and environmental DNA (eDNA) are more expensive and time consuming to process compared to invasively-collected samples, since the DNA extraction procedure is typically more complex, with replicated analysis sometimes required due to low DNA quality and concentration (e.g. in order to reduce the allelic dropout in microsatellite analysis), and because of the presence of exogenous DNA. Moreover, such non-invasive DNA sources yield small quantities of DNA that are not suitable for some downstream applications such as single nucleotide polymorphism (SNP) array genotyping or whole genome sequencing in their standard form.

To address these problems, tailored methods are being developed to enable SNP genotyping, target DNA capture, restriction site-associated DNA sequencing (RADseq), mitogenome and whole-genome sequencing, and metabarcoding based on low-quality DNA sources (reviewed in Andrews et al.^40^, Hohenlohe et al.^41^). The methods that have been successfully applied in wildlife studies include microfluidic genotyping of degraded samples with reduced SNP panels^11^, genotyping of microsatellite loci in faecal samples with high throughput sequencing^12^, targeted sequencing of DNA from faecal samples^13^, methylation-based enrichment of vertebrate DNA from faecal samples with RAD sequencing^14,15^ and the application of eDNA barcoding to establish the distribution of an endangered species using soil samples, combined with individual identification through nanopore sequencing^16^. The development of these methods allows for considerable progress in the utilisation of non-invasive samples in genomic research. Still, the cost and workload required to carry out such analyses is considerably higher compared with the standard genomic methods applied to high-quality samples.

An alternative approach may be to utilise a non-invasive source that provides high-quality endogenous DNA of sufficient quality to be analysed using standard methods, without the need of enrichment/pre-processing procedures after DNA extraction. Saliva is very promising in this respect, given that saliva samples were shown to provide quality and yield of DNA comparable to blood samples, and produce high-quality genotyping outputs^3,4,17^. However, unlike other non-invasive samples, which may be obtained without any direct contact with the animals studied and with minimum interference with their behaviour, the existing methods of saliva collection providing a sufficient quantity for genomic research require an animal to be approached and handled, which is inconsistent with the principles of non-invasive sampling and impossible without extensive habituation. The alternatives include a combination of baits and porous/absorbent materials that capture saliva^18–20^, or collecting residual saliva from the target species left on partially-consumed food items^21^, but these methods typically yield low DNA concentrations that are insufficient for genomic analyses. Therefore, there is a need to develop a method of saliva collection that does not require an animal to be approached but allows for the collection of sufficient amounts of saliva to produce high DNA concentrations required for SNP array genotyping and whole genome sequencing.

Here we present a method of saliva collection for DNA analysis that does not require approaching the sampled animal. The method was developed for free-ranging domestic dogs (FRDs) based on the commercially available Performagene saliva collection kits (DNA Genotek, Canada). FRDs are a good model to test and validate methods of genetic sample collection for wildlife, because – being free-living domesticated animals – it is possible to both test them at a distance (hence mimicking what would be necessary for wild species) but also test them using more standardized methods involving contact with humans without running the risk of altering their natural behaviour. This allowed us to compare the method of remote saliva sample collection with a method recommended by the manufacturer of the Performagene kits, which involves placing an absorbent sponge in the dog’s mouth.

The aim of the current study was to develop and evaluate the efficacy of a hands-off saliva collection method which would deliver sufficiently high-quality samples to allow for genomic analyses. We thus collected saliva samples from our FRD either using the hands-off/remote method, or the Performagene manufacturer’s recommended procedure. We then measured the DNA concentration in all samples and performed genome-wide SNP array analysis, comparing results of the two methods at each stage. This allowed us to test the viability of the remote collection of high-quality non-invasive DNA samples.

## Materials and methods

### Ethical statement

Ethical approval was obtained from the Ethical committee at the Agronomic and Veterinary Institute Hassan II (Comité d’Éthique de l’Institut Agronomique et Vétérinaire Hassan II) in Rabat, Morocco (Protocol number: CESASPV_2023_05). For sample collection, dogs were unrestrained, their participation in the test was voluntary, and they were able to leave at any time. All procedures were non-invasive and in accordance with EU Directive 2010/63/EU. All methods are reported in accordance with ARRIVE guidelines (https://arriveguidelines.org).

### Subjects and study site

Data were collected between June 2022 and June 2024 in Morocco (Sous-Massa region) from a resident population of free-ranging dogs. Dogs in the area live in or close to human settlements as scavengers. Although they roam freely, they rely on human-provided food, categorizing them as street dogs^22^. Genetically, they resemble European mongrels rather than a mix of various breeds^23^; Pilot et al., unpublished data). This population is followed by a team of researchers from the Domestication Lab (Konrad Lorenz Institute of Ethology, University of Veterinary Medicine Vienna) in collaboration with Prof. Ikhlass El Berbri (Agronomic and Veterinary Institute Hassan II, Rabat, Morocco) and has been observed seasonally since 2016, with continuous monitoring of dogs in the area beginning in 2022.

A database of individually named dogs comprising photographs and short videos allowed their recognition over time. New dogs were added to the database if encountered at least three times within two weeks. Each dog was assigned a unique name and its sex, age, neutering status, body condition, and physical traits (e.g., tail length and shape, ear position, coat colour) were recorded. Although these dogs can be considered habituated to human presence, their level of approachability varies across individuals, with some allowing humans to touch them (e.g. pet them) and others being more shy, hence impossible to approach^24^. As the population is studied also with the goal of understanding variation in social behaviour towards humans, no habituation nor socialization procedures are applied, so as not to interfere with the natural behaviour of the study subjects.

461 samples were collected from 326 individuals (171 females and 155 males) using non-invasive saliva collection kits (Performagene PG 100, DNA Genotek, Canada) and DNA was extracted according to the manufacturer’s instructions. Twenty-eight individuals were sampled more than once, using different methods (see below).

### Apparatus

**Fig. 1:**
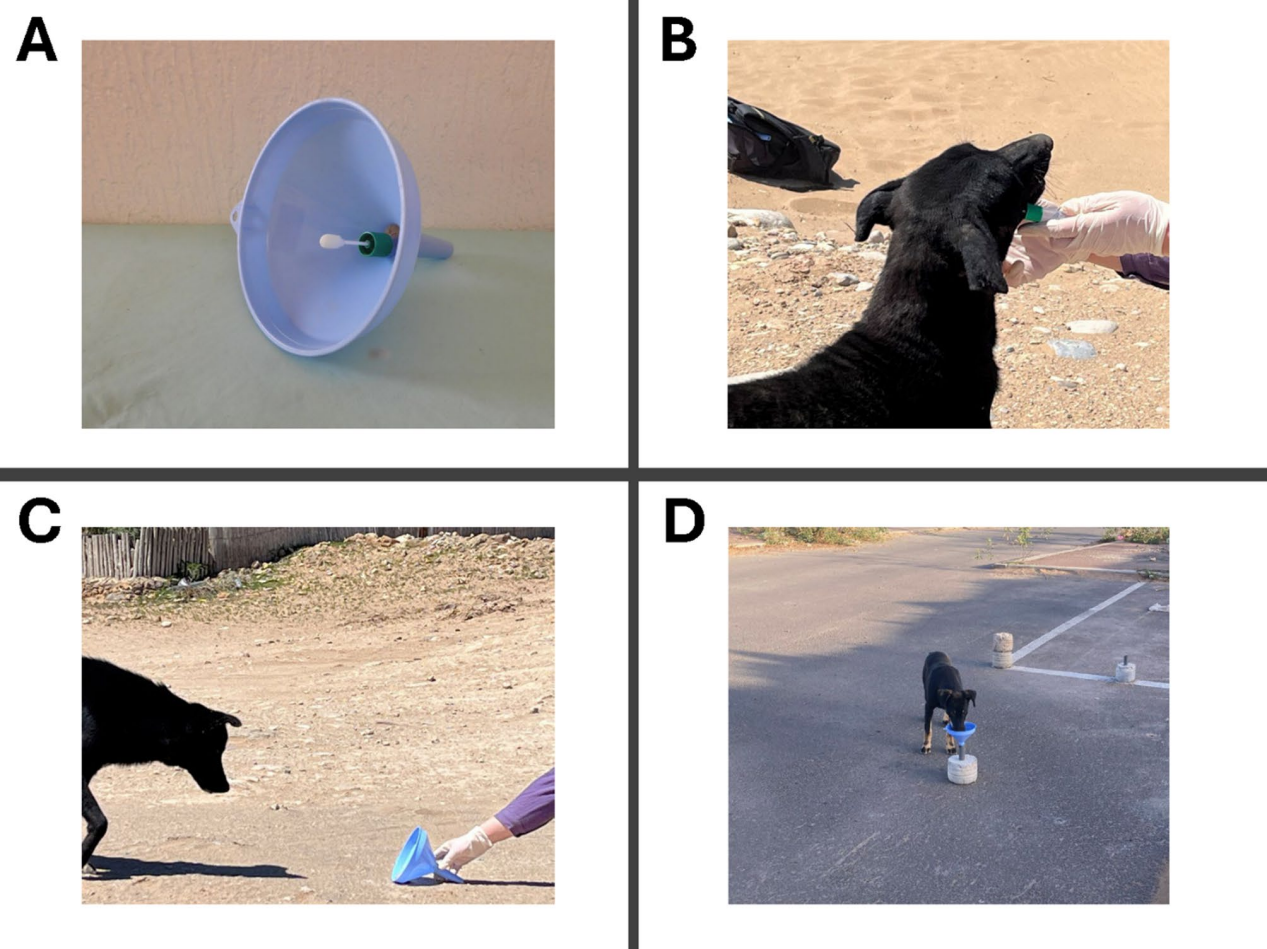
Saliva collection device (**A**); saliva collection method done by hand (hands-on), following the Performagene kit instructions (hands-on method) (**B**); using the device kept in the hand of the experimenter (held-funnel), **C**); using the device from a distance (ground-funnel: the device was opportunistically placed on an already existing structure present in the dog’s territory, **D**). Method (**C**) and (**D**) are collectively referred to as the “hands-off method”.

The hands-off device used to collect saliva samples from individual dogs was designed to accommodate different types of saliva collection kits. The apparatus consisted in a funnel made of plastic acquired locally. The wider, open top was 16 cm wide, while the small, tube-like opening at the bottom was 1.9 cm wide. The funnel had a height of 16.5 cm. One-two pieces of cooked or raw meat was placed in the tube-like opening to attract dogs and invite them to lick the swab. The meat was placed in a way that could be smelled but not reached. A saliva tube/swab was placed inside the tube-like opening in a way that allowed the swab to stick out of the tube, within the cone-shaped top, without having it touching the meat underneath. A metal wire passed through two holes at the top of the tube-like opening and used to fix the saliva tube/swab to the rest of the apparatus (Fig. 1A). The swab was carefully placed and fixed to the funnel so that it never got in contact with the meat underneath, nor with the surface of the funnel. Even when the apparatus was placed on the ground (Fig. 1C/1D), the swab could not touch the ground, even if the funnel was moved by the dog (Video S1). Disposable surgical gloves were used to manipulate the apparatus and were changed after each sample collection.

### Procedures—sample collection

Sample collection occurred opportunistically whenever a target dog was seen in the study area. Two-hundred-and-seventy dogs were directly approached by an experimenter who acted in a gentle, friendly manner, and samples were taken directly by hand, following the collection procedure recommended by the manufacturer of the Performagene PG 100 kits (hands-on method, Fig. 1B). A piece of meat was sometimes used as lure, but dogs were never fed before samples collection. Eighty-four dogs were sampled using the funnel device, which was either kept in the hand of the experimenter (held funnel; Fig. 1C) or placed on the ground (ground-funnel; Fig. 1D). Because preliminary analyses showed no difference in the quantity of DNA obtained from these two approaches, they were pooled together to test the general efficacy of the funnel collection device (hands-off method). In both cases, dogs were attracted by the presence of the meat and licked the swab placed on top of it, attempting to reach the meat underneath. After a few minutes, the device was retrieved by the experimenter and the swab/tube was closed following the manufacturer’s instructions. Whenever the swab entered in contact with something else other than the mouth of one dog, the sample was considered contaminated and discarded. Twenty-eight dogs were sampled more than once, using different methods (see below).

### DNA extraction and genotyping

DNA was extracted following Performagene protocol, with minor changes. DNA was eluted in water and extractions were performed using half of the sample and reagents volume. This allowed the extraction of the total sample in two steps: the first extraction for all the samples, and the second one for the samples that did not reach the 800 ng required for genotyping during the first extraction attempt, and called here “re-extraction”. For each extraction session, a negative control sample was used to control for external contamination during the extraction process. The extracted DNA concentration was measured using fluorescence-based Qubit HS dsDNA Assay (ThermoFisher Scientific), and absorbance spectrum checked using NanoDrop spectrophotometer (ThermoFisher Scientific).

Re-extracted samples with DNA quantity lower than 800 ng were added to the first extract. Good quality extracts with DNA quantity higher than 800 ng were genotyped using the Axiom Canine Genotyping Array (Thermo Scientific). Genotypes were called using Axiom Analysis Suite software and all the samples with more than 15% missing data were considered as failed.

### Comparison of saliva collection methods

Statistical tests were performed using R version 4.4.1. For each sample, the quantity of extracted DNA was calculated as the product of DNA concentration and elution volume. Extraction and re-extraction were considered as distinct data points.

First, we compared the DNA yield for the individuals collected with either the held-funnel or ground-funnel method with a Wilcoxon test. Because no difference was observed between these two methods, we pooled their results (i.e., hands-off method) to compare them with the hands-on collected samples. We tested if the DNA concentration was dependent on the saliva collection method, using a linear model with the amount of DNA as response variable and saliva collection method (hands-on vs. hands-off), the type of extraction (first extraction attempt or re-extraction) and its interaction with collection method as explanatory variables. Significance of each variable was tested using a type II F test from the *car* package^25^. To get closer to the normality assumption, the quantity of DNA (once added 0.0001 unit) was box-cox transformed using the *powerTransform* function from the *car* package.

Then, we focused on 28 individuals sampled several times, using both hands-on and hands-off methods. For each individual, the averaged value for the quantity of extracted DNA from hands-on or hands-off collection was computed. The number of individuals with a higher quantity of DNA value for hands-off than hands-on collection method was compared to the number of individuals with the opposite trend. The effect of collection method on the transformed values for the amount of extracted DNA was tested using individuals as random variable. The linear mixed model was fitted with *lme4* package^26^ and tested with *car* package.

Finally, we tested if the success of genotyping was dependent on the saliva collection method. A Pearson’s chi-squared test was used to compare the frequency of failed and successful genotyping, depending on the saliva collection method.

### Genotyping accuracy assessment

Genotyping of non-invasively collected samples is associated with two types of errors: allelic dropout, i.e. lack of amplification of one of the two alleles in heterozygous individuals, resulting in false homozygosity, and false alleles, i.e. amplification of additional, non-existing alleles, resulting in the overestimation of heterozygosity^27,28^. We genotyped the samples using the Axiom Canine Genotyping Array, which was developed based on prior knowledge of point mutations occurring in canine genomes, therefore it is unlikely that any of the two alleles identified in a given locus is a false allele. Allelic dropout is possible, although less likely than in the case of microsatellite loci, which were the markers of focus for most studies of genotyping errors associated with non-invasive genetic sampling^29^.

We could not assess the genotyping errors directly, because we did not have the genotypes of the same individuals based on samples collected using both the hands-on and hands-off method. Instead, we applied two indirect methods. First, we compared the levels of heterozygosity per locus between samples collected with hands-off versus hands-on approach. The proportion of heterozygotes per loci for the hands-off group was calculated using Plink v.1.9 software^30^. To control for the sample size, we carried out random subsampling from the hands-on group, using the same number of individuals as in the hands-off group, which was done in 10 replicates. Levels of heterozygosity for each locus were compared between these 10 random subsamples and the hands-off group. Decreased heterozygosity in the samples from the hands-off group compared with hands-on subsamples may indicate the allelic dropout.

To compare the overall numbers of genotypic errors between the two groups of samples, we assessed the number of mismatching loci between parent-offspring dyads. Mismatching loci are defined as the loci where a parent-offspring pair does not share any allele, and they reflect genotyping errors. We identified all the parent-offspring cases in our dataset using pi-hat value (the proportion of alleles with identity-by-descent among the genotyped autosomal SNPs) and z-scores obtained from the Plink v.1.9 software, using the following criteria: pi-hat > 0.49, z0 < 0.1, z1 > 0.8, z2 < 0.2, where z0, z1 and z2 are the proportions of loci with 0, 1 or 2 alleles, respectively, shared by the parent-offspring pair. We then used the CERVUS software^31^ to identify the number of mismatching loci for each parent-offspring dyad. The CERVUS analysis was carried out for 1100 SNPs, which were selected to maximize heterozygosity, because this software could not process a larger number of loci. The difference in the number of mismatching loci between the two groups was tested using a Wilcoxon test.

## Results

### The quantity of extracted DNA is higher when saliva is collected by hand

750 DNA extractions were performed on 461 saliva samples belonging to 326 different individuals.

The DNA concentration measured in extracts ranged from 0ng/ul to 836ng/ul, with an average value of 30.9 ng/ul (± 73.7, Table 1). Twenty-one extracted samples (10 collected by hand and 11 collected with the hands-off method) were below the detection limit from the Qubit (“too low” for measurement) and assigned to 0 ng/ul. The corresponding DNA quantity for all concentration measurements ranged from 0 ng to 83,600 ng, with an average value of 1393 ng (± 5604, Table 1).

**Fig. 2:**
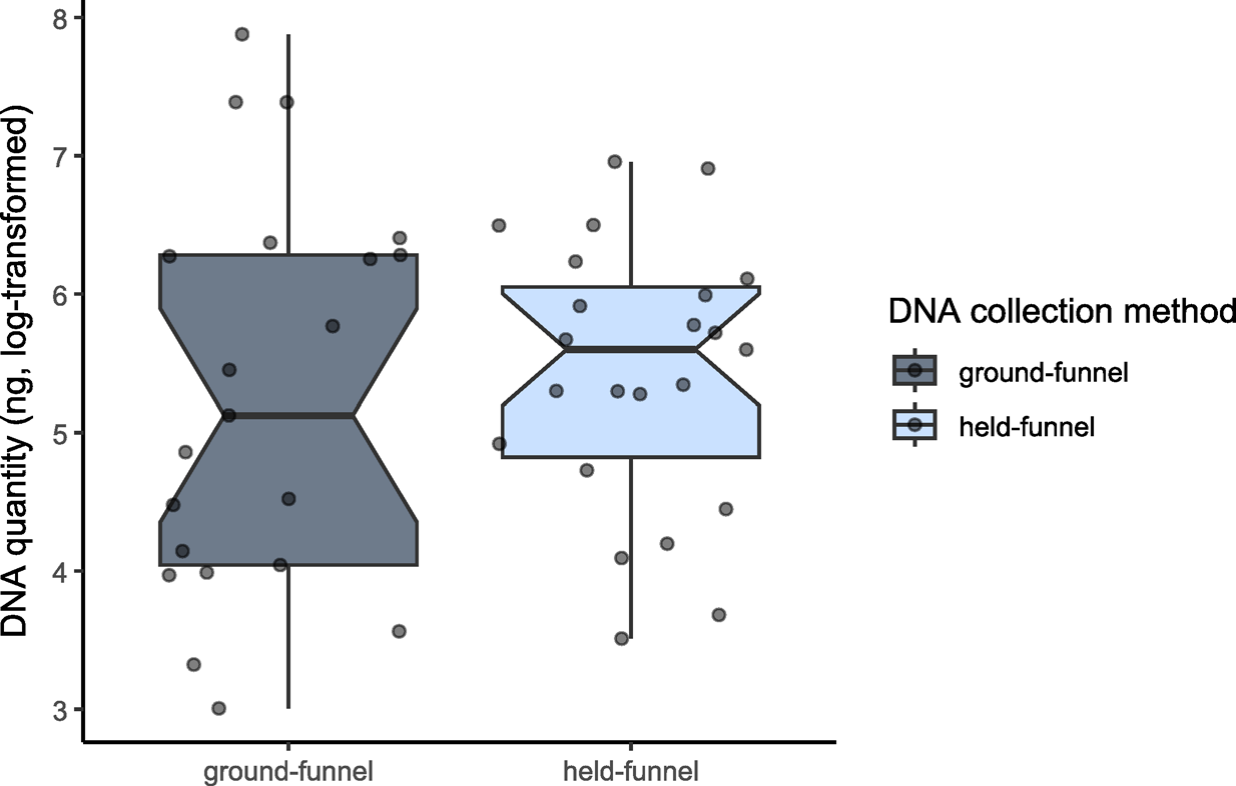
Quantity of DNA extracted from the held-funnel method and the ground-funnel method.

The different approaches used to collect saliva from the funnel device (held-funnel vs. ground-funnel) did not affect the DNA quantity (Wilcoxon test: W = 222, *p* = 0.489, Fig. 2).

**Fig. 3:**
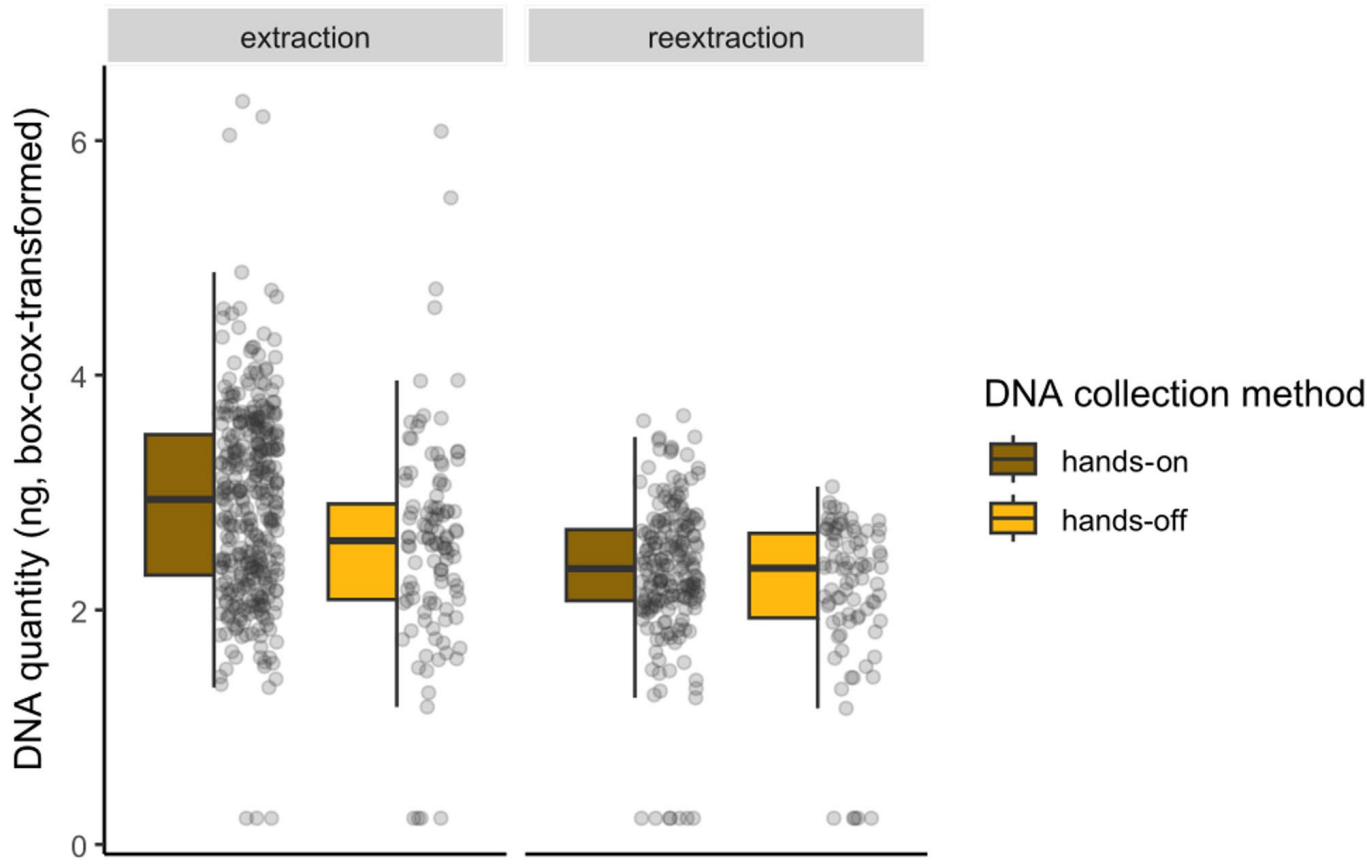
Quantity of extracted DNA depending on collection method and the type of extraction (first attempt: “extraction”, or second attempt: “re-extraction”).

The quantity of extracted DNA was higher when saliva was collected manually (hands-on method) compared with the hands-off method (F = 16.364, *p* = 5.771e-05, Fig. 3). While the amount of extracted DNA was lower for re-extraction compared with the first extraction attempt - this last category including individuals with a high quantity of extracted DNA and thus not re-extracted (F = 86.08, *p* = 1.832e-19, Fig. 3) - there was no interaction between the type of extraction and saliva collection method (F = 2.02, *p* = 0.156).

**Table 1:** Summary statistics relating to DNA extraction, depending on sample collection method.

	Hands-on	Hands-off
DNA concentration (ng/ul)		
Extraction		
Mean ± SD (n = number of samples)	46.3 ± 92.6 (*n* = 353)	32.2 ± 86.0 (*n* = 108)
Range	0–836	0–650
Re-extraction		
Mean ± SD (n = number of samples)	13.0 ± 17.1 (*n* = 204)	8.3 ± 7.6 (*n* = 85)
Range	0–114	0–28.6
DNA quantity (ng)		
Extraction		
Mean ± SD (n = number of samples)	2156 ± 7014 (*n* = 353)	1767 ± 7234 (*n* = 108)
Range	0–83,600	0–65,000
Re-extraction		
Mean ± SD (n = number of samples)	358 ± 471 (*n* = 204)	232 ± 208 (*n* = 85)
Range	0–2850	0–944

For individuals sampled with both collection methods, the quantity of DNA extracted was not higher when collected by hand (hands-on method) than when collected with the hands-off method.

**Fig. 4:**
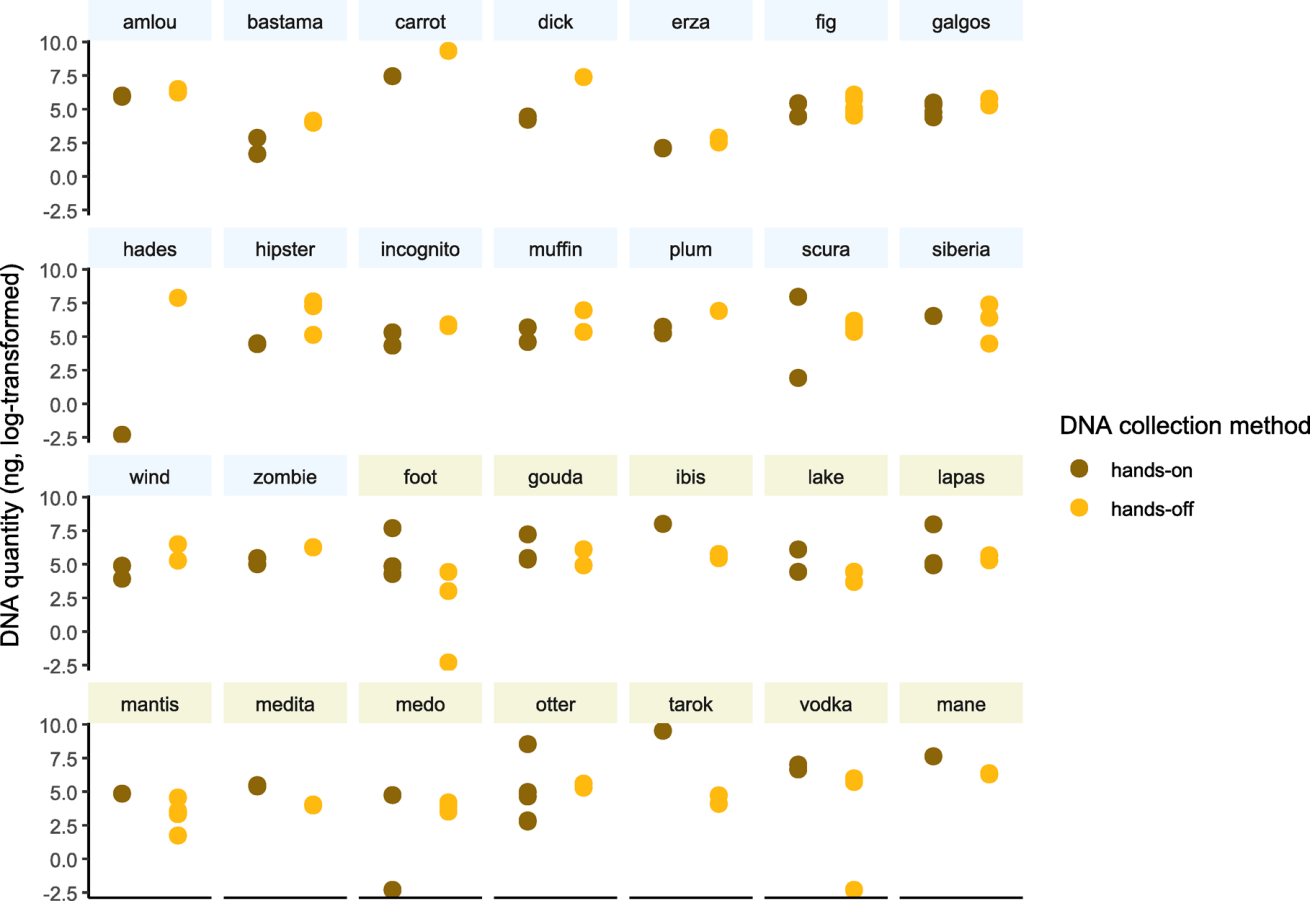
Quantity of extracted DNA per individual depending on saliva collection method. For the first 16 individuals (light blue labels), the quantity of extracted DNA was higher with the hands-off than with the hands-on collection method. For the last 12 individuals (beige labels) the quantity of DNA was higher with the hands-on than with the hands-off collection method.

28 individuals were sampled both with the hands-on and the hands-off collection method. For 16 of them (57%), the amount of extracted DNA for the samples collected with the hands-off method was higher than for the samples collected with the hands-on method (Fig. 4). For these 16 individuals, the hands-off collection method appeared to be more effective than the hands-on collection method. However, the quantity of extracted DNA was not explained by the collection method (F = 0.0001, *p* = 0.990).

### Genotyping success does not depend on saliva collection method

**Fig. 5:**
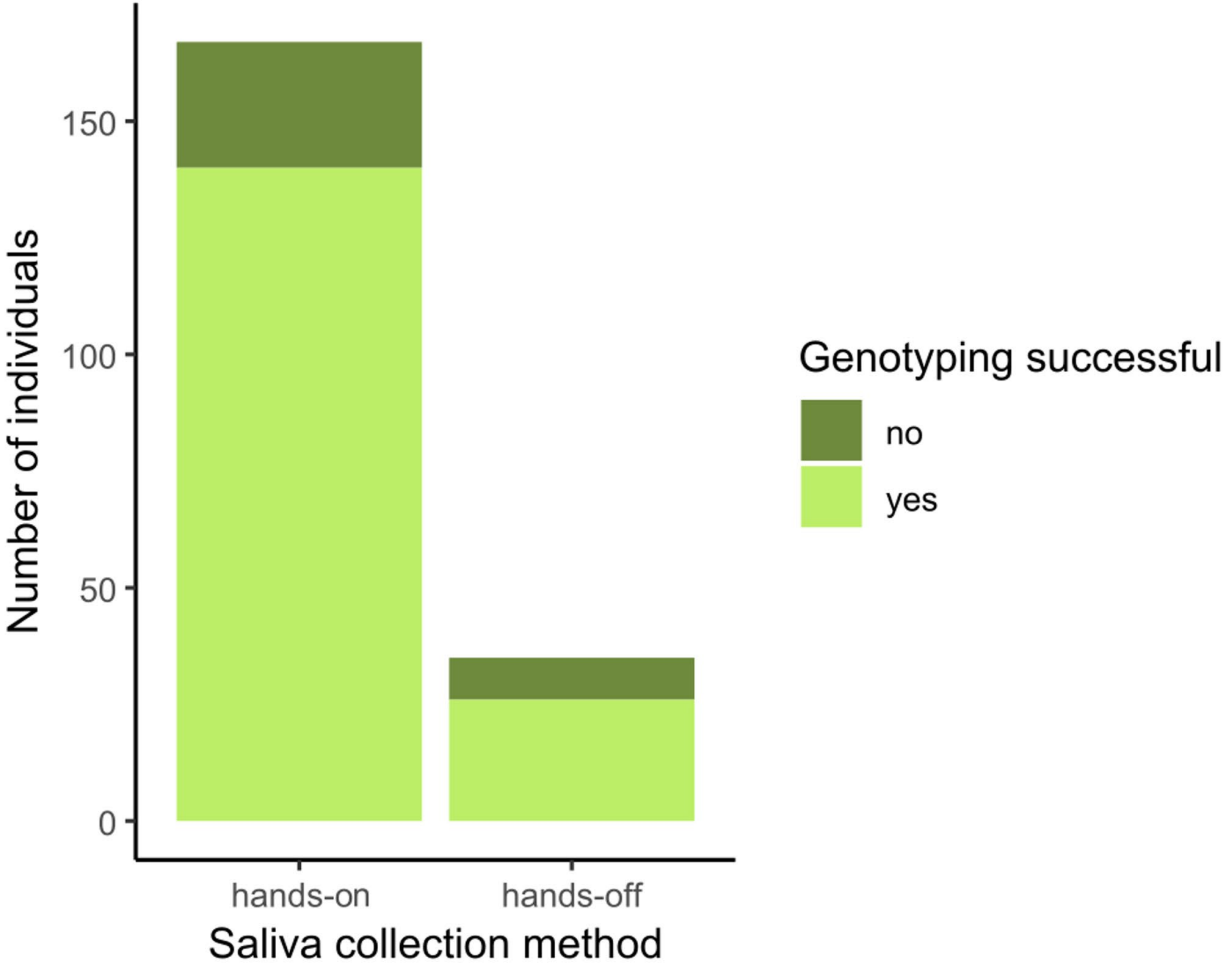
The number of individuals with successful or failed genotyping, depending on saliva collection method.

Of the 326 individuals included in this study, 202 were processed for genotyping and 37 of these genotyped samples were considered as having failed. Among the genotyped individuals, 26 were sampled with the hands-off collection method (16%). The sample collection method did not appear to have an effect on the genotyping success (chi-squared test: χ^2^ = 1.208, *p* = 0.272, Fig. 5).

### The genotyping accuracy is comparable between the hands-on and hands-off method

**Fig. 6:**
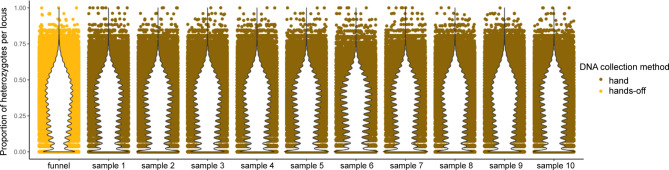
Distribution of heterozygosity levels in the group of samples collected with the hands-off method and 10 random subsamples from the group of samples collected with the hands-on method.

We compared the heterozygosity levels for each locus between the group of samples collected with the hands-off method and the 10 random subsamples from the group of samples collected with the hands-on method, with the same sample sizes (*N* = 26). The distributions of heterozygosity among the genotyped loci were very similar in each group (Fig. 6). The mean heterozygosity in the hands-off group (0.318 ± 0.184) was slightly higher compared with each of the 10 random subsamples (from 0.289 ± 0.183 to 0.310 ± 0.186), implying that the allelic dropout did not occur or was too rare to affect heterozygosity estimates. We also compared individual heterozygosity between the hands-off and hands-on methods and found no significant differences (Wilcoxon’s test: W = 1624, p-value = 0.41).

**Fig. 7:**
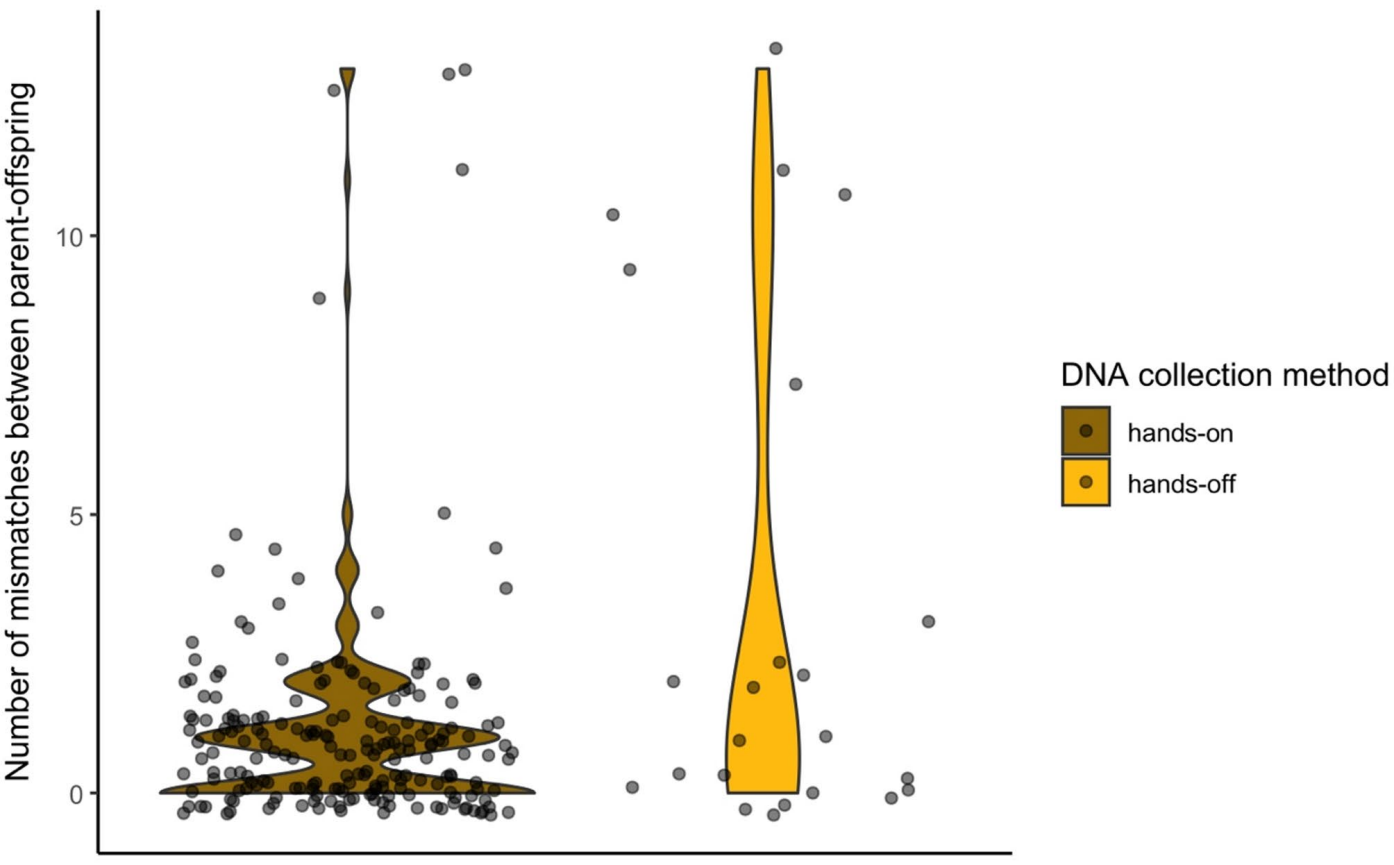
Distribution of the number of mismatching loci in parent-offspring dyads in hands-on and hands-off collection methods.

Among the successfully genotyped samples, 68 individuals with samples collected with the hands-on method and 13 individuals collected with the hands-off method had at least one parent-offspring relationship in our dataset. In total, we compared 202 parent-offspring relationships in the hands-on group with 23 parent-offspring relationships in the hands-off group, and found no significant differences in the number of mismatching loci (Wilcoxon’s test: w = 2731.5, *p* = 0.1429, Fig. 7). In both hands-on and hands-off groups, the number of mismatches ranged from 0 to 13 per 1100 loci, i.e. 0 to 1.2%, pointing to a high accuracy of genotypes generated based on the samples collected with both sampling methods.

## Discussion

The comparison of our hands-off method with the collection method involving direct contact with the dogs recommended by the manufacturer (hands-on method), showed that although our collection method gave significantly lower DNA concentrations, the samples that yielded sufficient DNA for the downstream genetic analyses provided high quality genotypes based on genome-wide SNP array. The estimated genotyping error rates were no higher than in the samples collected with the hands-on method and not exceeding 1.2%. We thus demonstrate the viability of the hands-off collection of high-quality non-invasive DNA samples that can be used for genomic analyses.

Until relatively recently, the majority of studies involving domestic dogs have been conducted on pet/family dogs or shelter dogs^32^, however in more recent years, from a variety of perspectives, free-ranging dogs have been recognized as an important, if understudied group^33^. Genetic studies of such populations have clearly demonstrated that they are not ‘abandoned’ pet populations, but rather, populations of free-living, free-breeding dogs that have lived alongside humans for millennia^23,34^. Genetic studies of such populations can enhance our understanding of the history of domestic dogs^23,34^, how they have adapted to their specific geographic environments^35^, and potentially reveal important aspects relating to gene-behaviour interactions^33,36^. However, free-ranging dogs can include animals that are highly friendly with humans, and easily approachable, but also individuals unlikely to allow humans touching them if not after sedation. Our hands-off method was designed to allow for the DNA collection also from such individuals, thereby reducing the potential biased sampling of bold individuals.

A number of other saliva sampling methods have been developed, which however fell short on this particular aspect. For example, Montgomery et al.^37^ developed a method involving a PVC tube containing a cotton rope imbued in vegetable fat allowing them to collect saliva for hormonal analyses in approximately 50% of the juvenile hyenas population under study. However, because the tube was handheld from the vehicle, and hence required the hyenas to approach, the researchers highlight that their collection was likely skewed towards bolder or more habituated individuals in their study population. Similarly, Higham and colleagues^38^ wrapped a cotton rope lightly painted with a sugary substance around an oral swab (Salimetrics), thereby enticing rhesus macaques to suck on both without contaminating the swab itself with the attractant. One end of the rope was held by the researcher, whilst the monkey sucked on the swab at the opposite end. This method yielded good results in relation to the hormonal analyses the researchers aimed to obtain, however, whereas 100% of infants approached and engaged with the swab, only approximately 50% of adults were successfully sampled. This suggests that, like the hyena study, the sampling method was biased towards bolder or more habituated individuals.

The current hands-off method adopts some of the same principles used above, for example separating the attractant from the swab, thereby reducing the chance for contamination. However, it adds to these by developing a simple collection tool, which can be presented at greater distance from the researchers, by attaching the device to a tree, or any other element in the animal’s environment (see Fig. 1C). It can hence be employed with habituated populations that will not tolerate close vicinity to humans, but that can still be monitored from a distance (e.g. from a car). A prior habituation process, involving placing food at decreasing distances from the apparatus, until the animal is comfortably taking food from it, may also be easily implemented in order to further increase the probability of sampling also those individuals that may show more neophobic tendencies.

Overall, the yield of DNA extracted from saliva collected with our hands-off device is significantly lower than that obtained with the method recommended by the manufacturer. However, the two methods are similar in terms of the quantity of extracted DNA when individuals sampled using both methods are compared. While we did not test for differentiation in behavioural traits between these individuals and other sampled dogs, this result may indicate that the collection method does not impact DNA yield for individuals which were bold enough to be sampled with the hands-on method at least once (and thus be approached and handled by a human), or for individuals that are not so neophobic as to avoid interacting with the funnel device at least once. This suggests that the difference in DNA yield is not solely dependent on the characteristics of the apparatus but also on the behavioural traits of individuals, which might affect the sampling success (e.g. some individuals can be sampled with both or only one of the two methods).

To our knowledge, the only other hand-off method to collect DNA from saliva in dogs was developed by Lobo and colleagues^18^ and tested on shelter dogs. Researchers presented shelter dogs with approx. 6 cm diameter cylinders of three porous substrates (wood, cork and Styrofoam) covered in bait (either sardine paste or canned dog meat). After eating the bait, dogs spent some time chewing or licking the substrate and then abandoned it, at which point the researchers collected and processed the items to extract the saliva. Results showed that chewing time and substrate type, specifically wood, were positively associated with saliva quantities, but bait type had no effect. Regardless of substrate type, mean DNA concentration was 14 ng/ul (corresponding to a DNA quantity of 700ng, as they used 50ul of elution buffer); with 75% of values under 10ng/ul (i.e., 500 ng), researchers report values being on a par with other non-invasive methods such as faeces^18^. With our method, the yield of DNA recovery is more than twice higher (mean DNA quantity of 1767ng using the hands-off collection method; but 75% of values under 700ng) and can be doubled with the use of the whole sample and reagent volumes. Nevertheless, from a logistics perspective the porous substrate with bait around it would need considerable adjustment for field work, requiring a means to tie the object in place, to avoid animals running off with it. Because that method^18^ involves a substrate already covered with potential contaminants (meat or sardines), this limits the use of DNA to genetic markers specific to the targeted species. Furthermore, the cleaning and saliva extraction procedure from the substrate requires additional resources which our method avoids.

Nevertheless, some limitations remain, the most prominent being that the researcher’s presence is still required in order to guarantee retrieval of the swab before a second animal, or other disturbance, contaminates the sample. In fact, despite this procedure could be applied to numerous species where individuals might tolerate the presence of humans within a certain distance, it cannot be used with more elusive species. Thus, the next step will be to modify the collection method building a remote apparatus ensuring that the collection medium (the swab), once used by a single individual becomes (1) unavailable for use by others and (2) remains protected within a container until the researcher can retrieve it. A similar system has been tested with small mammals^39^ where the sampling device disappears into a tube once the animals stops engaging with it, allowing the collected DNA to remain protected from contamination. The system depends on a pully and magnet system that keeps the collection medium available to the animal until it engages with it, but that becomes detached once the animal lets go of it, allowing for the retraction of the sampling medium into a tube.

In conclusion, in the current study we developed a hands-off DNA collection method using the popular Performagene sampling kit. The method was shown to be as efficient as the recommended hands-on method of collecting saliva from domestic dogs, and it was shown to be highly suitable for sampling free-ranging dogs. We also highlighted that the method led to highly accurate genotypes, with small genotyping error rates, which were no higher than those estimated for the genotypes generated for samples collected using the recommended hands-on method. The method can be applied also to numerous other wild and domestic species as long as the population is sufficiently habituated to the presence of a human/vehicle, allowing the researcher to retrieve the swab once it has been used by a single individual, and before potential contamination by other factors. Future modifications to render the system completely remote will need to consider mechanisms which will allow the swab to be protected after single use, and alert the researcher that retrieval is required.

## Supplementary Information

Below is the link to the electronic supplementary material.

Supplementary Material 1

## Data Availability

Data available from the Figshare Digital Repository (https://doi.org/10.6084/m9.figshare.28640156): Dataset with extraction information and R-script for all the analyses and figures are provided. For open access purposes, the authors (Giulia Cimarelli, Martina Lazzaroni, Clément Car, Ikhlass El Berbri, Sarah Marshall-Pescini, Malgorzata Pilot) have applied a CC BY public copyright license to any Author Accepted Manuscript version arising from this submission. All photos and videos depict and were taken by the research team.
